# Impact of pretransplant mutation status on survival after allogeneic stem cell transplant for acute myeloid leukemia

**DOI:** 10.1002/jha2.260

**Published:** 2021-07-22

**Authors:** William Selove, Lloyd Hutchinson, Vladislav Makarenko, Xiuling Meng, Keith Tomaszewicz, Muthalagu Ramanathan, Jan Cerny, Rajneesh Nath, Benjamin Chen, Bruce Woda, Jacob R. Bledsoe

**Affiliations:** ^1^ Department of Pathology UMass Memorial Medical Center University of Massachusetts Worcester Massachusetts USA; ^2^ Department of Hematology‐Oncology UMass Memorial Medical Center University of Massachusetts Worcester Massachusetts USA; ^3^ Department of Hematology Medical Oncology Banner MD Anderson Cancer Center Clinic Gilbert Arizona USA; ^4^ Bristol Myers Squibb Company Cambridge Massachusetts USA

## 1 INTRODUCTION

Allogeneic stem cell transplant (Allo‐SCT) is a powerful tool in the treatment of acute myeloid leukemia (AML) and offers the only potential chance of cure. Crucial to the success of Allo‐SCT is the elimination of leukemic blasts prior to transplant. Evaluation of residual disease historically has been via morphologic review, with complete remission defined as fewer than 5% blasts [1]. Although thresholds lower than 5% are prognostically significant [2], morphologically it is usually impossible to distinguish normal regenerating bone marrow blasts from leukemic blasts.

Distinction of pathogenic from regenerating blasts has been achieved via flow cytometry and by PCR or cytogenetic studies in cases with recurring genetic abnormalities. Unfortunately flow cytometry is plagued by lack of standardization and the requirement for analytic expertise for interpretation, especially in the context of AML residual disease monitoring. AML demonstrates vast phenotypic and genotypic heterogeneity, and FISH and PCR are applicable only in those cases with detectable genetic abnormalities, limiting the utility of these modalities. AML is also temporally heterogeneous, as the dominant phenotypic and genotypic features present at diagnosis can change significantly over the course of disease. This makes residual disease testing at times a “moving target.”

Next generation sequencing (NGS) offers a number of advantages for residual disease testing. NGS allows detection of numerous mutations across a broad array of commonly altered genes in a single assay. In the context of residual disease testing for leukemia, it allows detection of dominant and subclonal mutations both at diagnosis and in posttreatment monitoring and has the ability to detect molecular aberrations suspicious for persistent leukemia at low levels and across the gamut of subtypes.

The sensitivity of NGS for detecting residual AML and the prognostic significance of post‐induction residual low‐level leukemia‐associated mutations has previously been established by others. [3] Jongen‐Lavrencic et al showed specifically that residual mutations in genes other than *DNMT3A, TET2*, and *ASXL1* detectable after induction confer worse post‐induction survival.[3 ,4]

In the current study we endeavored to assess the significance of persistent AML‐associated mutations in the pre‐Allo‐SCT setting since relatively few studies have examined their significance in this context. We explored the impact of mutations present at diagnosis that remained detectable within 30 days of transplant on overall survival and disease‐free survival. Prior studies have suggested that mutations in genes involved in modification of the epigenome ‐ either through DNA methylation regulation or histone modification ‐ tend to be permissive of AML transformation but not sufficient in and of them to give rise to leukemia. [3, 5] Therefore, we focused on the implications of mutations in epigenetic modifiers, specifically ***I*** *DH1/2*, ***D*** *NMT3A*, ***A*** *SXL1*, ***T*** *ET2*, and ***E*** *ZH2* versus others (the acronym ***IDATE*** will be used to refer to these epigenetic modifier genes henceforth in this paper). We sought to explore whether the survival impact of these mutations purported to be merely “preleukemic” would be as significant as mutations in other classes of genes. We also evaluated the stability of these epigenetic modifier mutations by comparing their tendency to be eradicated through pretransplant conditioning (also known as rate of clearance) versus other pathogenic mutations.

We performed a retrospective analysis of patients who received Allo‐SCT for AML between 2009 and 2015 at the University of Massachusetts Medical Center. Thirty‐eight patients had mutations discovered in their diagnostic bone marrow specimens either by NGS or by PCR fragment analysis for *NPM1* or *FLT3* mutations. Seventeen patients had bone marrow samples available for NGS testing from both the time of diagnosis and within 30 days prior to transplant.

Diagnostic and pretransplant bone marrow specimens were sequenced via the CTMP version 3, 42 gene myeloid panel. Somatic mutations in diagnostic specimens were called based on tier 1 (pathogenic) or tier 2 (likely pathogenic) mutations at variant allele frequencies (VAFs) ≥ 5%. In pretransplant specimens molecular residual disease was defined as ≥ 0.2% VAF.

*NPM1* and *FLT3* mutation status was tested in the diagnostic specimens of all patients using PCR fragment analysis. In those patients with either mutation detected at diagnosis, supplemental PCR testing for these common insertion mutations was repeated in the pretransplant specimens.

We evaluated overall survival and relapse free survival according to the presence or absence of morphologic and molecular residual disease. We categorized residual mutations according to type/affected pathway (e.g., epigenetic vs. signaling, etc.). Mutation status at diagnosis was compared to mutation status immediately before transplant. Overall survival and relapse free survival were compared across mutation class categories with particular emphasis on epigenetic modifier mutations versus all others using the Kaplan‐‐Meier method and the log‐rank test. Overall survival was censored for patients lost to follow‐up. Relapse free survival was censored for patients that were lost to follow‐up or who died without diagnosed relapse. We also examined clearance rates of somatic mutations by subtype (using chi‐square test with Yates correction) to determine which mutations showed the greatest propensity to persist through pretransplant conditioning (i.e., remain detectable in the window between the end of chemotherapy and the initiation of transpant).

Patient median age at diagnosis was 59 (range 27–80), M:F 1.8:1 (Table 1). Thirteen patients showed normal karyotype, two had disease defining translocations, and two had other cytogenetic abnormalities. Five patients had myelodysplasia‐related changes. In pretransplant specimens, five patients had biopsies deemed positive/suspicious for persistent disease based on morphology. The other 12 patients were morphologically negative in their pretransplant specimens. Mutations identified at diagnosis included *FLT3, NRAS, PTPN11, KRAS, JAK2, NPM1, RUNX1, DNMT3A, IDH1/2, ASXL1, EZH2, TET2, SRSF2, SF3B1, SETBP1, WT1*, and *STAG2*.

**TABLE 1:** Patient characteristics

				Findings in diagnostic specimen				Findings in pretransplant specimen					
	Case	Age	Sex	Karyotype	Blast count (%)	Mutations	Median mutation percentage	Blast count (%)	Mutations	IDATE mutation only	Median mutation percentage	Relapse free survival in months	Overall survival in months
Pretransplant marrow containing at least one non‐IDATE mutation	1	66	M	inv(3) ‐	14	NRAS, NPM1	33	10	NRAS	N	8	2.1 +	2.1
	2	57	F	46, XX	83	NPM1, DNMT3A, IDH2, PTPN11	46	40	NPM1, DNMT3A, IDH2	N	43	6.1+	6.1
	3	67	M	46, XY	34	NPM1, PTPN11	39	8	NPM1	N	3	1.3+	1.3
	4	81	M	'‐7, +r(7),	94	JAK2, IDH2, RUNX1, EZH2, FLT3‐TK, PTPN11	56	30	JAK2, IDH2, RUNX1, EZH2, FLT3‐TK, RUNX1	N	27	6 months	9.4
	5	64	M	46, XY	83	NPM1, IDH1, SRSF2, NRAS, PTPN11	42	5	NPM1, IDH1, NRAS, IDH2	N	29	4.0+	4
	6	77	M	46, XY	80	NPM1, DNMT3A, SF3B1, SETBP1, TET2, NRAS, KRAS, PTPN11	47	<5	FLT3‐ITD, NPM1	N	1	0.4 +	0.4
	7	43	M	inv(9)	92	FLT3‐ITD, NPM1 DNMT3A IDH2	46	0	NPM1, DNMT3A, IDH2	N	0	2.6	9.5
	8	73	M	46, XY	97	FLT3‐ITD, NPM1, DNMT3A	45	0	FLT3‐ITD, NPM1, DNMT3A	N	4	3.0 +	3
	9	36	F	t(6;9) (p23;24)	20	FLT3‐ITD, NRAS	24	3	FLT3‐ITD	N		12+	12
Pretransplant marrow showing IDATE only mutations	10	68	M	46, XY	62	ASXL1, DNMT3A, IDH1	21	6	ASXL1, DNMT3A, IDH1	Y	44	62.2+	62.2+
	11	35	M	46, XY	97	NRAS, DNMT3A, IDH1, BCOR, STAG2	48	0	DNMT3A, IDH1	Y	5	12.4	23.3+
	12	56	F	NP	74	FLT3‐ITD, NPM1, DNMT3A	58	0	DNMT3A	Y	1	36.8+	36.8+
	13	77	M	46, XY	NA	FLT3‐ITD, NPM1, TET2	33	0	TET2	Y	24	2.6+	2.6
	14	80	F	46, XX	67	FLT3‐ITD, DNMT3A, RUNX1, IDH2	45	<5	DNMT3A	Y	40	54.9+	54.9+
Pretransplant marrow showing no mutations	15	59	M	46, XY	25	NPM1, NRAS, IDH2	42	2	None	–	0	56.2+	56.2+
	16	44	F	46, XX	76	FLT3‐ITD, RUNX1, WT1	46	<5	None	–	0	23.4	26.5
	17	27	F	46, XX	NP	NPM1	40	1	None	–	0	55+	55+

Abbreviations: N, no; NP, not performed; Y, yes; +, censored (lost to follow‐up for OS; lost to follow‐up or death without relapse for RFS).

Of the 17 patients with mutations detected at diagnosis and a pretransplant specimen available for analysis, 82% (*N* = 14) showed persistent mutations in their pretransplant specimens (Table 1). Notably, many of the persistent mutations were in genes encoding proteins involved in epigenetic modification including the DNA methylation modifying proteins *DNMT3A, TET2*, and *IDH1*/2 and the chromatin modifying proteins *ASXL1* and *EZH2* (here termed “***IDATE”***). Among this 14 patient subgroup, 36% (*n* = 5) showed persistent mutations ***only*** in genes coding for proteins involved in epigenetic modification (IDATE) while the other 64% (*n* = 9) showed persistent mutations in ***at least one*** non‐epigenetic modifier (non‐IDATE) gene. Eighteen percent (*n* = 3) showed no residual disease in their immediate pretransplant specimens either morphologically or by molecular studies.

At diagnosis, of 56 total mutations identified among all patients, 27 mutations were cleared through pretransplant conditioning (Table S1). Broken down by class of mutation, 16% (3/19) of epigenetic modifiers (IDATE) were cleared, while 61% (11/18) of class 1 (signal transduction) mutations were cleared, 50% (7/14) of class 2 (differentiation) mutations were cleared, and 100% (5/5) of other classes of mutations were cleared. Epigenetic modifier mutations showed a significantly higher propensity to persist (not to clear) in the pretransplant specimen than all other types of mutations combined (*p* = 0.001.)

**FIGURE 1:**
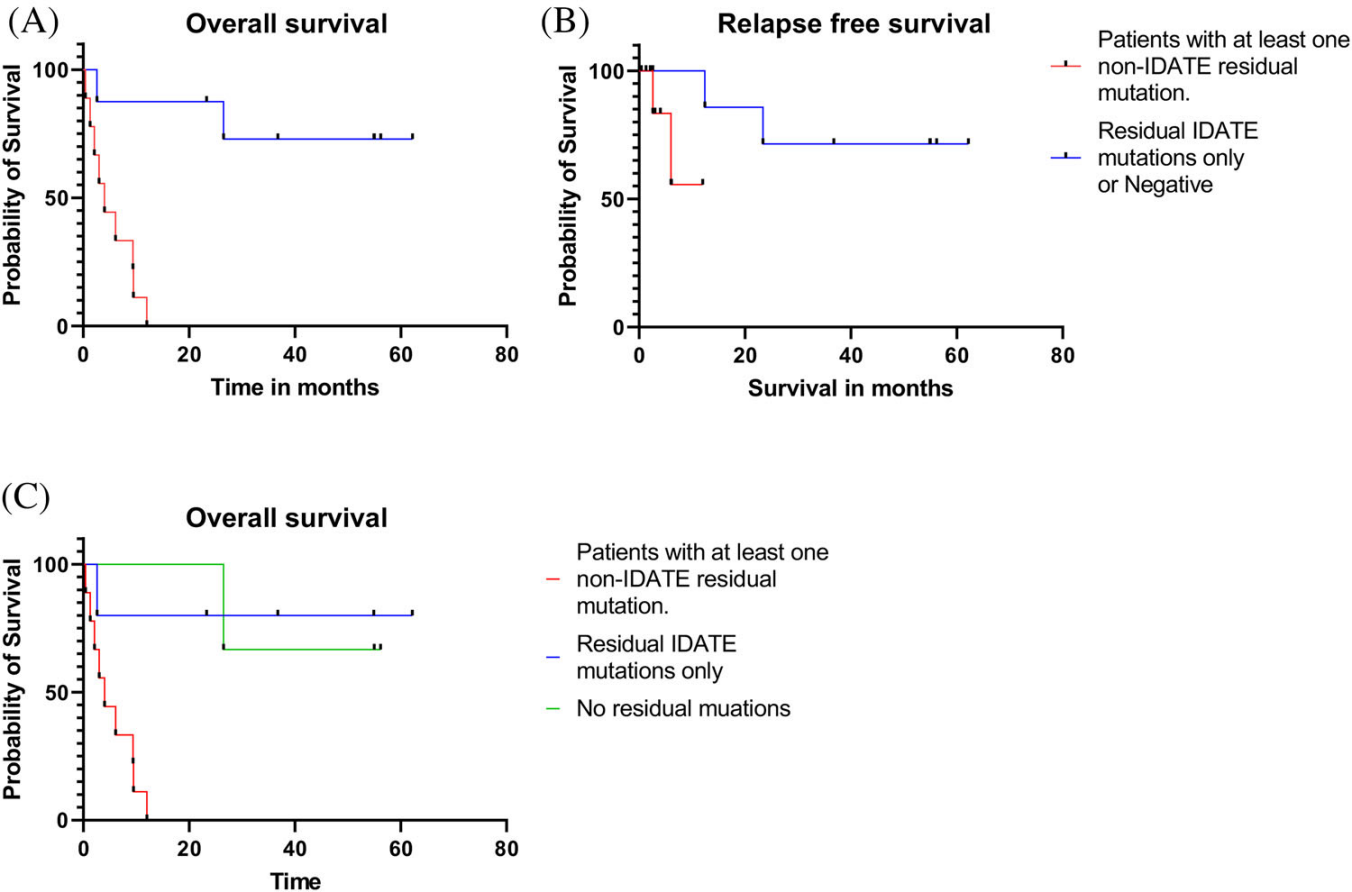
Posttransplant survival based on pretransplant mutation status. Patients with only residual IDATE or no mutations in pretransplant specimens had a superior overall survival (A) and a trend toward superior relapse free survival (B) in comparison to patients with at least one residual non‐IDATE mutation. Patients with only residual IDATE mutations had overall survival similar to patients in whom no residual pretransplant mutations were detected (C)

Patients with residual mutations in at least one non‐IDATE gene showed significantly poorer overall survival (HR 8.50, *p* = 0.0005) (Table 2, Figure 1A) and a trend toward poorer relapse free survival (HR 4.49, *p* = 0.059) (Figure 1B) than patients who had no residual mutations other than IDATE. Among residual mutation positive patients specifically, patients with non‐IDATE persistent mutations showed poorer overall survival than those with IDATE only mutations (HR 8.98, *p* = 0.0087) (Table 2, Figure 1C). The prognostic impact of non‐IDATE residual mutations on overall survival was also seen when analysis was restricted to morphologically negative patients. In fact, median survival for morphologically negative patients with non‐IDATE mutations was even shorter (5.8 months) than those with ostensible morphologic residual disease (16.2 months). This unexpected finding can probably be attributed in great part to the interesting case of patient number 10 who showed 6% blasts in his pretransplant bone marrow , technically constituting morphologic residual disease but who showed only IDATE mutations by NGS. The patient lived 62 months before being lost to follow‐up.

**TABLE 2:** Survival impact of pretransplant residual mutation status

Mutation Status	Hazard ratio (log‐rank)	*p*‐value
Overall survival		
Any mutation vs. molecular negative	3.82	0.15
Non‐IDATE vs. IDATE or negative	8.5	0.0005
IDATE only vs. molecular negative	0.78	0.86
Non‐IDATE vs. IDATE	8.98	0.0087
Non‐IDATE vs. molecular negative and IDATE among morphologically negative patients.	6.68	0.0056
Leukemia‐free survival		
Non‐IDATE vs. IDATE or negative	4.49	0.059

Our results show that molecular minimal residual disease (MRD) in non‐IDATE genes (non‐ *IDH1/2, DNMT3A, ASXL1, TET2*, and *EZH2*) in bone marrow specimens biopsied within 30 days prior to ASCT predicts worse outcome in AML compared to IDATE mutations. Persistent mutations in epigenetic modifiers (IDATE, sometimes referred to as class 3 mutations) have relatively little impact on posttransplant survival compared to mutations in other classes of genes. Epigenetic modifier mutations also show less susceptibility to clearing through pretransplant conditioning.

These findings are consistent with previous studies that have suggested that epigenetic modifier mutations as a class seem to be merely pre‐leukemic rather than true drivers of AML. [5, 6] It is well‐established that mutations in epigenetic modifiers (particularly *DNMT3A, TET2*, and *ASXL1*) are frequently present in preleukemic states such as clonal hematopoiesis of indeterminate potential [7]. Although they confer greater risk for leukemic transformation, they are not strongly blast‐associated. This is in contrast to mutations such as *NPM1* which, when present, have in prior studies proven to show stable associations with blast count and relapse [8, 9, 10].

Although the prognostic implications of residual mutations in AML patients post‐induction in genes other than *DNMT3A, ASXL1*, and *TET2* have previously been demonstrated,[3] there have been relatively few studies that have examined the significance of NGS‐defined residual disease in the pre‐stem cell transplant setting. Getta et al established an increased risk of posttransplant relapse in patients with NGS‐detected mutations in pretransplant specimens within 30 days prior to stem cell transplant. [11] Thol and colleagues showed both diminished overall survival and relapse‐free survival in patients with NGS‐defined pretransplant MRD other than *DNMT3A* and *NPM1*. [12] In a recent paper Press et al similarly established the prognostic impact of pre‐stem cell transplant MRD on posttransplant leukemia‐free survival in their cohort [13]. However, none of these studies focused on the specific survival implications of non‐IDATE mutations versus IDATE mutations. The current study lends further evidence for the unfavorable outcome predicted by persistent mutations prior to Allo‐SCT, but it also specifically helps to confirm that the preponderance of added risk is attributable to non‐epigenetic modifier (non‐IDATE) mutations.

Although our investigation produced significant results, it is limited by relatively small sample size necessitating a focus on classes of mutations rather than individual genes. Additionally there was some disparity among patient and tumor characteristics and among pretransplant conditioning regimens in our cohort. Larger studies with the statistical power to delve with greater granularity into the prognostic impact of individual mutations (with a particular focus on IDATE vs. others) and to adjust for varying patient, tumor and treatment characteristics are merited.

## CONFLICT OF INTEREST

Dr Jan Cerny serves in the advisory board/consultancy for Jazz Pharmaceuticals and Amgen. He is a Data and Safety Monitoring Board Member for AlloVir, and holds stocks from Actinium Pharmaceuticals, Bluebird Bio Inc., Dynavax Pharma, Atyr Pharmac, Gamida Cell, Miragen Therapeutics, Mustang Bio, Novavax, Ovid Therapeutics, Sorrento Therapeutics, TG Therapeutics, Vaxart Inc, and Veru Inc., outside the submitted work.

## Supporting information

Supporting Information
