# Supplementary material for: Impact of pretransplant mutation status on survival after allogeneic stem cell transplant for acute myeloid leukemia
Source: EJHaem. 2021 Jul 22;2(3):514–9. doi: 10.1002/jha2.260 (PMC9175694; doi:10.1002/jha2.260)
Supplement: Supplementary file 1 — Supporting Information [file JHA2-2-514-s001.doc]

| Table S1: Mutation clearance in pre-transplant specimens | | | | |
| --- | --- | --- | --- | --- |
| **Gene** | | | **Mutations present at diagnosis (n)** | **Mutations cleared (n, %)** |
|  | Class 1 (Signal Transduction) | |  |  |
|  |  | FLT3 | 8 | 4 (50) |
|  |  | NRAS | 4 | 2 (50) |
|  |  | PTPN11 | 4 | 4 (100) |
|  |  | KRAS | 1 | 1 (100) |
|  |  | JAK2 | 1 | 0 |
|  |  | Total | 18 | 11 (61) |
|  |  |  |  |  |
|  | Class 2 (Differentiation) | |  |  |
|  |  | NPM1 | 12 | 6 (50) |
|  |  | RUNX1 | 2 | 1 (50) |
|  |  | Total | 14 | 7 (50) |
|  |  |  |  |  |
|  | Epigenetic | |  |  |
|  |  | DNMT3A | 8 | 1 (13) |
|  |  | IDH2 | 5 | 1 (20) |
|  |  | IDH1 | 3 | 0 |
|  |  | TET2 | 2 | 1 (50) |
|  |  | ASXL1 | 1 | 0 |
|  |  | EZH2 | 1 | 0 |
|  |  | Total | 19 | 3 (16) |
|  |  |  |  |  |
|  | Other | |  |  |
|  |  | SF3B1 | 1 | 1 (100) |
|  |  | SRSF2 | 1 | 1 (100) |
|  |  | SETBP1 | 1 | 1 (100) |
|  |  | WT1 | 1 | 1 (100) |
|  |  | STAG2 | 1 | 1 (100) |
|  |  | Total | 5 | 5 (100) |
